# Notes of Cases in Surgery

**Published:** 1862-02

**Authors:** Frederic C. Skey

**Affiliations:** Surgeon to St. Bartholomew’s Hospital


### NOTES OF CASES IN SURGERY.

By FREDERIC C. SKEY, Esq., F. R. S.,

*Surgeon to St. Bartholomew's Hospital.*

The leisure hours preceding the commencement of the ensuing medical session enable me to recall to my recollection some of the more remarkable examples, whether of disease or of injury, that have fallen under my charge during the past year in the wards of St. Bartholomew's Hospital. Of these cases I propose to give a general sketch, in the hope that they may prove not unacceptable to some of my professional brethren who, by the exigencies of practice or the remoteness of their locality, are deprived of the advantage of an occasional visit to the wards of an hospital.

### *Fractured Bone.*

I commence the task I have assigned myself by referring to the cases of fractured bone, of which the supply to my own wards has been amply sufficient to meet the wants of the most earnest students of his profession. Three hundred and twenty-one cases of *fracture* in general—of which nineteen were compound—have been admitted in the thirteen weeks in which I have been on duty. The single week at Christmas last produced no less than forty seven cases. Three cases of compound fracture appeared to me to demand amputation. In several others the injury was great: some to muscles, while in some the bone was greatly comminuted. In four or five, hæmorrhage followed either primarily or secondarily.

I quote one case of some interest, arising from the great extent of the injury, which was far greater than would twenty years since have warranted the decision arrived at:

A man, aged forty-six, was admitted in December last with extensive laceration of muscles and integuments of the lower end of the right forearm and comminuted fracture of both bones, caused by machinery. The question of amputation was one of difficulty, arising from the extent and complication of the injury. I confess I was influenced by the fact that the man was the father of a large family, and that the loss of his right arm would be fatal to his occupation. He was a healthy man, of abstemious habits; and, moreover, I entertain a strong impression that the amputation of a limb—certainly of a forearm—may be undertaken with equal, if not greater, safety at the expiration of a few days as at the date of the accident. We have yet something to learn on the subject of "primary amputation." The forearm was "put up." Slight hæmorrhage from the radial artery occurred on the seventh day, and profuse on the ninth, when the artery was tied. The man was greatly reduced by the loss of blood, his vital powers being sustained for several days by brandy, in half-ounce doses, frequently repeated; the quantity determined by the pulse. The man was discharged in the twelfth week from the date of his admission, with a limb sufficiently restored to its natural functions for the resumption of his pursuits as a printer.

In weighing the question of amputation of a limb for serious injuries, there is something beyond the amount of damage done to be taken into our calculation of the probable future. I allude to the subject of constitutional health, whether primary, or determined by the habits or occupation of the individual. These are questions not readily determined on early inquiry, and some days may elapse before the real power of the individual to avert or contend against disease is ascertained by the surgeon. The vital powers may suddenly fail at the time they are most required. I lost two cases of compound fracture of the leg, in which the extent of injury was far less in degree than several others which ultimately recovered. It may be said, I should have done well had I at once amputated these limbs. The result proving fatal, doubtless the operation of immediate amputation would have presented an alternative, possibly a more favorable one, but this *ex post facto* reasoning is of little value when placed by the side of general principles, which proclaim aloud the paramount duty of the surgeon to save, and not to destroy.

Simple fractures of the leg I have treated commonly by means of the application of a large pillow whenever the displacement has been moderate. When great, or the fractures very oblique, with a tendency to retraction, I have resorted to the cradle with side splints, a positive objection to which is the liability to protracted ulcerations of the heel.

Of fractures of the leg of the more ordinary kind, some 35 cases have recovered thoroughly well by the application of the pillow, which is an efficient and by far the most comfortable agent I know in the treatment of this injury, a well-made flock pillow will answer this purpose well. It should be of length sufficient to reach above the knee, and extend some inches below the foot. When bound on to the leg by means of four or five straps with buckles, which are drawn very tight, the pressure, as is well known, is diffused over the entire surface of the leg, and it is unproductive of pain or of such an amount of discomfort as to prevent sleep on the first night following its application.

### *Strangulated Hernia.*

The cases of strangulated hernia have presented in about the average number in the year, and in their arrival they have observed the same irregularity which has so frequently excited observation from others—viz: to their appearing in groups, of two or three within so many days. I do not know that I have ever operated on two cases of hernia identical in character. Each case has its own peculiarities, presenting features distinct from any other. Where such features are so peculiar as to involve the question of treatment, or to determine the course of operation required for it, the examples claim attention as cases of more than usual interest, and of such I shall refer to three.

The first is that of an old woman, aged eighty-eight, admitted in May last. She was the subject of double hernia, femoral on the right, and inguinal on the left side. The right presented a hard and painless swelling, which, according to the old lady's positive declaration, had existed in its present condition for many years. It gave her neither pain nor discomfort. The left swelling was large, soft, and painful. Examination by the hand was invariably followed by sickness and by shooting pain into the abdomen. In fact, she referred her own symptoms exclusively to the left side, declaring as she pressed her hand on the right swelling that it had nothing to do with her present malady. I thought I succeeded in returning a portion of the mass into the abdomen; but the symptoms continued, and I decided to operate. The woman being placed under the influence of chloroform, with the concurrence of my colleagues, Messrs. Lawrence, Stanley, and Savory, I exposed the mass on the left side. The sac contained omentum only, and a part of which was readily returned through an opening which admitted a finger without effort. The right swelling was left untouched; this decision being arrived at on the positive assurance again and again given by the patient that she had never suffered a moment's pain or inconvenience from the swelling in the right groin, and also from the probability that intestine had been returned, before the operation. The symptoms, however, continued unrelieved; vomiting and constipation were as urgent as before, although her constitutional powers appeared little reduced by the previous discipline. On the following day I operated on the hard and insensible swelling in the right groin. On opening the sac, a quantity of venous blood escaped. A large knuckle of small intestine formed the mass of the swelling, which was highly congested with blood, and was shut off from the abdomen by a very slight stricture, which was divided with some difficulty. The peculiar features in this case are—first, the double hernia; secondly, the peculiar sympathy manifested by the stomach, as the dominant organ of the abdomen, with the tumor on the left side where there existed a strangulation; and thirdly, the entire absence of physical pain connected with the femoral hernia of the right side. Although the sickness and vomiting were no doubt maintained by the condition of the strictured intestine of the right side, yet the immediate relationship between the stomach and the tumour of the left side was so striking as to attract the observation of my colleagues as well as myself. This fact, the entire absence of local pain on the right side, and the woman's repeated request that we should "leave the right side alone," decided the question of the operation to be selected. At the time, the evidence appeared good, so far as it went; but it is quite obvious it was unsound at bottom, and led to a false diagnosis.

The second case was that of a man, aged thirty-eight, admitted in July last. He was a man mentally characterized by a low order of intellect, and incapable of a connected statement of his symptoms. It was extracted from him, with some difficulty, that he first became the subject of hernia twelve months prior; that the hernia returned without effort on his part on lying down; that he had a second attack six weeks previously, which also yielded to simple pressure and position; and that his present condition had existed consequent on a third attack of three or four days. He was visited by a medical man, who administered a full dose of calomel-and-colocynth, and leeched the swelling freely, but without apparent benefit. On inquiry, we ascertained that he had been the subject of a discharge from the urethra, and had passed his urine with difficulty. The course of a catheter was obstructed at two inches from the orifice of the urethra. The local condition was as follows: Right inguinal canal much swollen; left slightly so; scrotum very largely but uniformly distended with fluid; penis involved in the general swelling; perinæum also greatly enlarged; constipation of four days, but no abdominal pain; violent sickness of greenish matter; pulse intermittent. Thus were presented, with tolerable distinctness, groups of symptoms of two diseases bearing no relation to each other. The aspect was that of extravasation of the urine; the symptoms of those of strangulated intestine. It is true that the swelling of the right groin belonged to the *canal*, and not to the *subcutaneous tissues;* and this fact formed an important element in the diagnosis. On the one hand we had obstinate constipation, the evidence of previous rupture, distension of the inguinal canal, and violent and scarcely remitting sickness; on the other, stricture, impermeable to a small catheter, with urethal discharge and local appearance which we are far more accustomed to identify with extravasation of urine than with hernia. The *history* was that of hernia; the local *aspect* that of extravasation. There could be little doubt of the presence of hernia, whereas that of extravasation was more uncertain, and of the two diseases less critical. I operated for hernia. The sac was in a condition of large and general suppuration, the effects of which had involved all the surrounding parts, scrotum, penis, and perinæum. The intestine was marked by numerous gangrenous patches. I selected one of the smaller of these, and punctured it with a trocar, when a large quantity of purulent fluid escaped. I divided a tight stricture, tied up the aperture with a fine silk thread, and returned the intestine into the abdomen.

A woman aged thirty-nine, three months advanced in pregnancy, admitted in May last, fifteen hours previously was seized with sudden pain and a swelling in the right groin, consequent on an attempt to support a child at arm's length; vomiting quickly followed. The right groin, on admission, was the seat of a small hard knot, on which no impression could be made by the hand. I operated within two hours after her arrival, and on opening the sac nearly two pints of watery fluid, as near as we could calculate, escaped from the peritoneal cavity. The intestine, which was gorged, but not unhealthy, was returned into the abdomen; vomiting ceased. The main feature in this woman's case consisted in the discharge of an immense quantity of fluid, of a yellow color, and containing flocculi, through the wound from the abdominial cavity. The bowels acted freely on the third day. On the fifth she died. No post-mortem examination was permitted. It was not, however, very essential to the explanation of the cause of death.

One word on the subject of the division of the sac in cases of hernia. The operation of non-division has, I confess, disappointed me; and I am disposed to concur with many other authorities in the belief that the advocates of the operation are not borne out in their estimate of its supposed advantages; such, at least, is the result of my own personal and necessarily limited experience in the practice of St. Bartholomew's Hospital. The operation of strangulated hernia, although the theory is obvious enough, and the object to be obtained clearly understood, is often an operation of considerable difficulty and of anxiety to the surgeon operating; and in a large number of cases a young surgeon is not indisposed to suspend for the moment his "pride of place," and to avail himself of the advice and co-operation even of his assistant, certainly of his seniors, should such be present. I consider the difficulty of the operation, as a rule, considerably increased by the attempt to divide the stricture from without inwards; or, in other words, without opening the sac which surrounds the intestines.

### *Dislocation.*

Of thirty-five cases of dislocation, a few only have fallen under my own immediate charge. I may allude to one example of dislocation of the femur occurring in the person of a stout boy of sixteen. The head of the bone was thrown backwards on the dorsum acetabuli, and it resisted the combined force of four persons engaged in extending the limb while the boy was under the influence of chloroform. On remitting the extension, the bone was reduced by bending the thigh forwards on the pelvis and drawing it outwards. Of seventeen cases of dislocation of the humerus, in one only was the head of the bone thrown on the dorsum scapulæ. The above list includes a case of dislocation of the coccyx, some particulars of which may be worthy of notice. The subject was eminently sensitive and hysterical young woman, aged twenty, who sustained the injury by a fall in the street three weeks prior to her admission into the hospital. At the date of the accident she was too far advanced in pregnancy, and she miscarried in the internal. Examination detected displacement of the bone, which was thrown forwards at a right angle with the sacrum, the point projecting against the rectum. Tolerably firm pressure made through the bowel readily replaced it, but it returned to its abnormal position immediately on the remission of the pressure.

My first intention was to operate on the bone from within, and with this view I designed a metal spring, as thin in the neck as was compatible with strength, to be introduced into the rectum; but the difficulty of fixing it to the plevis, and of employing the exact force requisite, was insuperable; besides which, I had no experience as to the capability of the bowel to bear the required pressure for many days, and probably weeks. I then determined on the experiment of direct traction of the bone, and, having cut down upon it, passed a thin silver wire around at about three quarters of an inch from its extremity. The wire broke, and I replaced it with a large silk thread, which was drawn tightly round the point of a broad wooden splint adjusted to the woman's back. The experiment, however, was not very successful; symptoms of very active hysteria manifested themselves, and she declared the pain to be insupportable. Some amount of local irritation certainly attended the progress of the case, with local redness and moderate suppuration, but not to an extent commensurate with her suffering. She wore the instrument twenty days, during the whole of which she declared the pain very severe. For a few days she appeared relieved; but the bone returned to its abnormal relation to the sacrum. Subsequently she became eminently hysterical, and even maniacal, and I was compelled to remove her from the ward. From this state she recovered; her condition gradually improved, and she left the hospital relieved from her symptoms, both local and constitutional.

My experience of the past year in reference to dislocation at and below the ankle-joint (which students yet recognise under the curious term, "dislocation of the *tibia*," and occasionally of the *leg*) confirms my former opinion that the foot may be dislocated *in any degree* and in any direction; but more especially does this remark apply to dislocation of the foot, both backwards and forwards.

*Lithotrity.*

Some cases of lithotrity that have fallen under my observation are not without interest.

A man, aged sixty-eight, admitted in November last, had suffered from symptoms of stone for three years, during which he had passed about one hundred small stones, varying in size from that of a large shot to that of a pea. I broke the stone by eight several operations, each of which was unproductive of serious pain, and was followed by more or less relief. A small fragment only remained in the bladder. After the last operation, which was not characterized by any remarkable feature, he had rigors, vesical pain, and low fever, and he died. The post-mortem examination revealed disease of long standing in both kidneys.

A boy, aged fifteen, admitted in October, had had symptoms of stone for two years. I crushed the calculus, which I presumed to be about the size of a large almond, twice only, at an interval of ten days. The boy left the hospital well.

A boy aged ten, admitted in January, had suffered from symptoms of stone for three years, during which he is reported to have had several attacks of retention of urine. His urethra being large, and readily dilitable, I decided on treatment by lithotrity, and for which I had a small lithorite made. The stone was crushed twice, without pain. On the following morning a fragment of stone became impacted in the urethra, and retention supervened. No alternative remained but lithotomy. As I was unable to pass in the bladder, I cut down on the urethra without it; and, having removed two large fragments of stone from the canal, I opened the bladder by the aid of a staff, and expelled the residue of the calculus by injection. More than half the stone had lodged in the urethra. The boy recovered. I freely confess the attempt to remove a stone from the bladder of a boy ten years of age by lithotrity to have been an error in judgment. The first fragment that escaped was of considerable size, and at once blocked up the canal. The rule I have consequently adopted for my own guidance is, never to operate with the lithotrite unless the urethra will admit a catheter of the size of No. 9 or 10. In this patient's case the lithotrite was No. 6, though the urethra was dilatable to about No. 7, or perhaps 8.

A man, aged seventy-two, was admitted in May, from whom I had removed a stone by lithotrity, in 1851. The peculiarities of this case were connected with the physical qualities of the stone itself, from which arose a very protracted term of treatment. I operated on him no less than seventeen times. The calculus was of lithic acid composition: and I may remark, after some considerable experience of the operation of lithotrity, that the hardest calculi I had ever broken have consisted of lithic acid, and not of oxalate of lime. I never once *crushed* this patient's stone; I only broke it into small fragments. No part of it was ever reduced to powder, as in other examples; but the entire stone, when finally collected, consisted of a multitude of fragments of nearly equal magnitude—about that of a large shot. The force of the screw required to break the stone continued very great up to nearly the last operation. On several occasions I came to apparently a dead-lock, and it required all the power at my command to screw the instrument home. Notwithstanding this force, at the termination of the case the blades of the lithotrite did not indicate more than a very slight strain in the workmanship, so perfect is the material of which these instruments are constructed.

There are, in my opinion, comparatively few cases of calculus in which the operation of lithotrity is not available. It may be most efficiently performed by any person possessed of ordinary skill and knowledge, and I earnestly join in its advocacy by the greatest of our modern surgeons.

*Burns.*

In reference to the subject of burns, I have always been an advocate for the stimulating principle of treatment first suggested by Dr. Kentish. That principle, to my observation apparently so sound, has been repudiated of late years by many eminent surgeons. The thoroughly negative action of Carron oil and lime water has been substituted for it, from which, I confess, I have never seen a corresponding benefit obtained. It is notorious that the intense pain caused by a drop of hot sealing-wax, or other similar agent, on the skin is much mitigated by the temporary application of great heat. The pain subsides in the course of a few moments by holding the affected part to the fire. The explanation is not perhaps very obvious, but the fact remain.

In July last five men were admitted into St. Bartholomew's Hospital, with severe burns on the face, head, chest and arms.

One died immediately after his arrival. My house surgeon, Mr. Richard Smith, was at the time ill, and the immediate charge of the cases devolved on a colleague, who applied the oil and lime water to the arms and hands of each of the surviving men. At that moment Mr. Smith arrived, and completed the dressing by the application of a solution of nitrate of silver (ten or twelve grains to the ounce of water) to the face, neck, etc. I did not see these men until the following morning, when all four complained of severe burning pain in the arms and hands, but stated they were free from pain in the other affected parts. The stimulating solution above mentioned was applied to the upper extremities, and the relief at the expiration of about a quarter of an hour was complete. In two of the cases some pain returned on the third day, and relief was obtained by the same means. My directions are simply these: In the case of infants or young children, wash the affected surface, if not very extensive, with a solution of nitrate of silver, in strength six or eight grains to the ounce, and immediately cover up the part with a thick mass of cotton wool; in the case of an adult, from twelve to fifteen grains, unless the surface requiring [illegible] application be very large. Should pain return, the solution may be advantageously resorted to at an early stage of the treatment.

*Varix.*

Some twenty-five cases of varicose veins of the leg have been treated in all my wards, and all by the application of the Vienna paste, composed of two-fifths potassa fusa, and three-fifths quick lime. The results have not shaken my confidence in the efficacy of the treatment. The eschars can scarcely be made too small, provided the material of which it is composed be thoroughly good and recently mixed. The numbers vary according to the extent of the disease. From twelve to eighteen will suffice usually for a limb somewhat largely affected. As regards the period of time required for the after treatment, it is of course of no great moment whether the number of the eschars be five or twenty-five, as they all progress towards recovery at the same time. I have never yet seen any evil consequence, either local or constitutional, arise from the application of Vienna paste to the largest and most prominent vein, however thin may be the textures over it. The cure is generally, but not invariably, permanent.

*Housemaid's Bursæ.*

I have purposely admitted a large number of cases of what are termed "housemaid's bursæ," and they may be supposed to have presented themselves in every varity of form, character and stage of progress. I am not sanguine enough to entertain the hope of shaking the confidence of many surgeons in the efficacy of blisters and tincture of iodine, which may be applied and reapplied for months without the smallest impression on the disease, so far as I have witnessed their effects. Indeed, with respect to the latter agent, I regret to say that I know of no chemical or therapeutical agent in such general resort as a local remedy in disease which is so commonly inoperative for good; and in these bursal diseases it appears to me to be especially so. If a full-sized thread be passed through the centre of the swelling, be it large and hard, giving the sensation of a solid mass, but in the centre of which is always found a small cavity of fissure; or be it soft, and containing fluid, whether large or small, suppuration in the course of from two to five or six days will inevitably follow. The thread may then be removed. The disease is converted into an abscess, and may be treated as an abscess. I may assert, without exaggeration, that I have cured from one hundred to two hundred cases on this simple principle. No other caution is necessary beyond the removal of the thread when the orifices through which it has passed indicate the inflammatory action incidental to its presence.

The same agent, and on the same principle, is equally applicable to *ranula.* Indeed it is quite remarkable with what rapidity this disease recedes under the action of the thread, whether the cyst be of average or of the largest size. Of the latter I have reported some, and treated several of such magnitude as to require the lower end of the thread to be brought out in the neck at some distance below the base of the jaw.

### *Hœmaturia.*

I have treated during the last year several cases of hæmaturia from malignant disease of the bladder. As a rule, I do think serious hæmorrhage from the organ to be a striking, and certainly not a permanent, feature of the disease. The early stage is marked by local pain arising from the cavity of the bladder, and frequent micturition. As the disease advances, dull pains are referred to the sacrum and posterior aspect of the pelvis. The introduction of a catheter, however carefully performed, is attended with more than the ordinary pain incidental to the operation, and is often very severe. On turning the instrument in the bladder, it will be observed to rotate more freely in one direction than in another, and its withdrawal is followed by bleeding, sometimes considerable, but often continuing or recurring at short intervals, for a day or night, or longer. Pain is caused by pressure on the hypogastrium, and through the rectum, but not on the perieneum, as is incidental to active disease of the prostrate. In some cases, however, the hæmorrhage is frequent and in large quantity, being either entirely mixed with the urine, or partially deposited in a clot at the bottom of the vessel. The bleeding will often cease for many days, or even weeks, and the pain becomes less considerable. Loss of appetite, impaired sleep, and emaciation follow, and inevitable death.

A man, aged thirty-nine, was admitted in November last, discharging blood mixed with his urine in large quantity. He had been the subject of disease for six months; had pain on pressure over the bladder; frequent micturition, but rarely without blood. He had no symptoms of renal disease. The introduction of the catheter gave great pain on reaching the organ, which, unlike ordinary catheterism, continued long after the withdrawal of the instrument. Occasionally the hæmorrhage would cease for many days, and the pain partially, and both would again recur without cause apparent to observation. He appeared for a time to be relieved by gallic acid and opium; but I really cannot say his occasional improvement was positively attributable to the treatment. He left the hospital in a weakly condition, and I shortly heard of his death, but his bladder was not examined.

A man, aged fifty, was admitted in November, with dull, aching pain in the bladder, and frequent micturition, from which he had suffered three or four months. On introducing a catheter, No. 6, very cautiously into his bladder, he expressed great suffering. The instrument did not move freely in the organ, and exhibited a tendency to fall over the right side. Blood escaped along the canal before the instrument was withdrawn; although no effort had been made in the introduction. Pain followed the use of the catheter, and continued for many hours. It was only arrested by an opium suppository. But the bleeding continued till the night of the day following. There was no evidence of stone. The hæmorrhage returned in a week, and continued in spite of astringents and opium for several days; but the pain was unremitting. In two months from this date he died. The left side of his bladder was occupied by a large growth of soft cancer.

I now leave the wards of St. Bartholomew's Hospital with a view to state the particulars of the case of a well known member of our profession, who died last winter. He was at the time I allude to (October last) under the care of an eminent physician, for pain referred to the descending colon, which he himself attributed to distension of the intestine; but he had also suffered for many months from frequent micturition and heat along the track of the urethra. I was requested to see him in reference to the subject of stone. I could not myself refer his symptoms to this cause; but as it was most desirable, in regard to his future treatment, that this question should be set at rest, I examined him. The introduction of No. 6 catheter, passed with the greatest care, was attended by very severe pain, extending along the lower part of the canal, but greatly aggravated when the instrument reached the prostrate gland. Here the instrument labored, as it were, as though the canal were either contracted in calibre, or distorted from the straight line. Immediately the catheter entered the bladder, the point was carried over to the right side, nor could it be held straight with the mesial line. Blood followed the withdrawal of the instrument, and slight bleeding continued for three days. About ten days afterwards I was sent for very urgently, and I found Dr. ——— in the water-closet, greatly alarmed, fearing to move. He told me he had had an attack of pain, of the severest endurable character, commencing in the left testicle, and extending along the cord to the bladder, where it continued with an intensity compared with which all he had previously suffered was trifling. A glass of strong brandy and water and an opium suppository completed the relief which had commenced before my arrival. Up to this date, therefore, the evidence stands thus: 1. Frequency of micturition for about three months. 2. Great pain on the introduction of a catheter, and difficulty of passing it through the prostate gland. 3. The instrument carried over to the right side of the bladder. 4. Hæmorrhage continuing for three days. 5. Sudden attack of intense pain in the testicle, cord and bladder. 6. The existence of cancerous disease in the person of a near relative. Diagnosis: Cancerous tumour of the left side of the bladder, involving the prostrate gland. No attempt was made to confirm this opinion by any repetition of the local examination. Dr. ——— lived about two months. The post-mortem examination revealed the presence of a large firm cancerous growth, of the size of the fist, occupying the left side of the bladder, which was pushed over to the right side, and involving the prostate gland, through which the urethra was much curved to the right side, and contracted in diameter.

### *Wounds into Joints.*

A few words on the subject of wound into joints. A boy, aged twelve, was admitted in July last, with a small wound on the inner side of his knee-joint. It was difficult to ascertain with accuracy the time or the nature of the cause, which, however, had occurred several days prior to his admission. Some four or five days afterwards, Mr. Smith abstracted a pin from the wound, which had obviously penetrated the joint, from which synovial fluid now escaped for several days. The fluid became purulent and copious; the joint hot, swollen and extremely painful. Matter collected under the skin in the neighborhood of the original wound, which required a second puncture. Large flabby granulations, almost invariably accompanying wounds into joints, formed around the two orifices, and existed for a month. The granulations at length subsided, the wounds healed, and the joint regained its normal size and appearance. Can this boy recover the former mobility of the joint, or is anchylosis inevitable? I believe it quite possible that the functions of the joint may be restored. I have had two cases of wounds into the knee-joint, in which discharge of synovia in many days' duration was followed by the free escape of pus from the cavity of the joint, continuing for a fortnight in one case, and ten days in the other; and in both cases the joint entirely recovered its functions. Of the restoration of the joint in the above case, however, I am not sanguine, from the protracted nature of the disease, the progress of which occupied a term of many weeks. The subject has not been investigated, as far as I know, but it is not devoid of surgical interest.

### *Breaking-down of the Joints.*

On the subject of breaking-down of joints fixed by deposit around them from rheumatic inflammation, I fear I have been less successful in my treatment than other members of the profession, judging from the published record of cases. But of the half dozen that have unfortunately fallen under my charge, in not one have I succeeded in restoring the functions of the joint, all having returned to their previous state of "spurious anchylosis."

### *Foreign Body in Pharynx.*

In June last I was summoned to a curious accident. A

young woman was admitted, wearing a set of artificial teeth, consisting, if I recollect right, (for I quote from memory) of four connected by a plate of metal, from each extremity of which descended hooked processes to fix it on the gum. The plate became detached, and she swallowed it, and it fixed across the pharynx about an inch from its termination in the œsophagus. The accident occurred in South Wales; and, having made one unsuccessful attempt to remove the foreign body, the medical attendant brought her to London. The accident had happened twenty hours when I saw her. The woman was in much distress, not from obstruction in the pharynx merely, but also from pressure on the larynx. The finger, passed with a considerable effort, reached the object, but only touched it with difficulty. From Fergusson's ample collection of forceps, of every form, size and variety, I succeeded in selecting a pair. After two or three futile efforts, having fairly grasped the foreign body, I abstracted it, not without the application of great force, so tightly was it held by the pharyngeal muscles. I do not pretend to any skill in the accomplishment of this not very difficult task, which owes its success to the advantage afforded by an hospital in selecting out of a large number the appropriate instrument for the purpose required. I kept the woman in the hospital till the following day, during which interval she exhibited no bad symptoms, beyond slight bleeding and soreness in taking food.

### *Hysteria.*

In concluding this somewhat lengthened communication, let me direct the especial attention of the younger members of the surgical profession to one of the most remarkable features associated with the treatment of disease. I allude to the great prevalence of *hysteria* in the cases of young women. It is quite remarkable how general is its presence, and how frequently it is interwoven with the symptoms of real disease. Abundance of cases recur to my recollection during the past year; reputed affections of the spine, and of the knee-joint more especially, at first sight of the most deceptive character. I would go so far as to assert that its presence, in some degree or other, is almost universal. And yet how critically important is a correct diagnosis in such examples! What form of treatment has a greater tendency to aggravate the symptoms of hysteria than that which may be judiciously applied to the cure of real disease?—*London Lancet.*

